# Playing Extensive Games with Learning of Opponent’s Cognition

**DOI:** 10.3390/s24041078

**Published:** 2024-02-07

**Authors:** Chanjuan Liu, Jinmiao Cong, Weihong Yao, Enqiang Zhu

**Affiliations:** 1 School of Computer Science and Technology, Dalian University of Technology, Dalian 116024, China; chanjuanliu@dlut.edu.cn (C.L.); cmj111@mail.dlut.edu.cn (J.C.); weihongy@dlut.edu.cn (W.Y.); 2 Institute of Computing Science and Technology, Guangzhou University, Guangzhou 510006, China

**Keywords:** sensor networks, intelligent sensors, games, adversarial learning, artificial neural network

## Abstract

Decision-making is a basic component of agents’ (e.g., intelligent sensors) behaviors, in which one’s cognition plays a crucial role in the process and outcome. Extensive games, a class of interactive decision-making scenarios, have been studied in diverse fields. Recently, a model of extensive games was proposed in which agent cognition of the structure of the underlying game and the quality of the game situations are encoded by artificial neural networks. This model refines the classic model of extensive games, and the corresponding equilibrium concept—*cognitive perfect equilibrium* (CPE)—differs from the classic *subgame perfect equilibrium*, since CPE takes agent cognition into consideration. However, this model neglects the consideration that game-playing processes are greatly affected by agents’ cognition of their opponents. To this end, in this work, we go one step further by proposing a framework in which agents’ cognition of their opponents is incorporated. A method is presented for evaluating opponents’ cognition about the game being played, and thus, an algorithm designed for playing such games is analyzed. The resulting equilibrium concept is defined as *adversarial cognition equilibrium* (ACE). By means of a running example, we demonstrate that the ACE is more realistic than the CPE, since it involves learning about opponents’ cognition. Further results are presented regarding the computational complexity, soundness, and completeness of the game-solving algorithm and the existence of the equilibrium solution. This model suggests the possibility of enhancing an agent’s strategic ability by evaluating opponents’ cognition.

## 1. Introduction

### 1.1. Background

Decision-making is a basic component of various agents’ behaviors [1], such as Autonomous driving and sensor systems [2], and has been studied in many fields such as psychology, economics, and artificial intelligence due to its ubiquitousness [3,4]. One’s cognition plays a crucial role in decision-making processes and outcomes, since available alternatives can be identified and weighed effectively only when meaningful information is collected. Cognition involves several aspects, including memory, learning and perception, and thus has attracted the interest of researchers in psychology, neuroscience, cognitive science [5,6,7], etc.

Focusing on mathematical analysis of interactive multi-agent decision-making processes, game theory has gained increasing acknowledgement as a classic tool in areas [8,9] including wireless networks, Blockchains [10], Robots, and so on [11]. The game-playing process can be significantly influenced by the player’s cognition regarding the possibilities of different choices and the suitability of these choices, since a player makes his/her choices based on such a cognition. To model and explain the various interactive decision-making scenarios in social and economic activities [12,13], different game-theory models have been studied. A typical game model is extensive games [14,15,16,17]; this model is used in sequential decision-making (SDM) scenarios [18]. In an extensive game, players take turns to choose actions; thus, a game tree is normally used to represent the process of an extensive game. In the game tree, each node represents a game situation, while each edge represents a move between game situations [19].

### 1.2. The Challenge

To find optimal solutions to extensive games, backward induction (BI) [20,21] is a well-known method. It computes backwards from the terminal nodes of a game tree to the root of the game tree. During this process, the player is assumed to be fully rational, always pursuing the most optimal choices by searching over the whole game tree. Consequently, the resulting solution concept via the BI algorithm is referred to as subgame perfect equilibrium (SPE) [22,23].

However, in the actual game-playing process, the limitations of computing power, memory, time, skills, etc., must be taken into account. Therefore, it seems impossible and unnecessary for the player to search the entire game tree, especially in large games. Instead, the player considers merely a portion of the game tree [24,25]. Meanwhile, based on prior knowledge, accumulated experience and playing tactics, the players hold their own opinions about the plausibility of future actions and the suitability of these game situations following the current decision point. Hence, both the classic model and the equilibrium for extensive games are too ideal to represent how the game is actually being played and the practical game-playing outcome. As a result, there is a need to develop alternative models and equilibrium concepts for extensive games, which should provide more realistic insights into the actual game-playing process.

Recently, [26] proposed a novel model of extensive games called extensive games with cognition, in which the agents’ cognition (including the underlying game being played and the quality of different game states) was simulated by artificial neural networks (ANNs) [27,28]. Unlike the standard CPE, the equilibrium concept under extensive games with cognition is dubbed cognitive perfect equilibrium (CPE). CPE seems to more accurately reflect the game-playing of players, and the ideal assumption regarding the visibility of the complete game tree to the players is weakened.

Despite the progress in the aforementioned framework, in which players’ cognition plays an indispensable role, essential work must be conducted in pursuit of modeling of practical game-playing. A key point is that the cognition in the existing model precludes the modeling of the opponent’s cognition. However, acquiring the opponent’s cognition of the underlying game would naturally benefit a player’s decision-making by allowing them to recognize their opponent’s strategy. This characteristic coincides with how humans play games, since they take advantage of their opponent’s expected reactions. Although the importance of reasoning about an opponent’s strategy was noted [29,30], not much attention has been paid to modeling game playing in consideration of an opponent’s cognition.

### 1.3. Our Contribution

In this paper, we build upon the model of cognitive extensive games and propose a model of extensive games with learning of the opponent’s cognition. The resulting equilibrium concept is called *adversarial cognitive equilibrium* (ACE). In contrast with SPE and CPE, ACE ignores the ideal assumption of full rationality by considering both the player’s cognition and his or her views on the opponent’s cognition. More specifically, we focus on the following issues:

(1) Modeling of adversarial cognition in extensive games. In Section 3, we propose a model of extensive games involving the opponent’s cognition, which we call *extensive games with adversarial cognition*, in which each opponent is endowed with his expected cognition on the game tree and the evaluation of game situations therein. First, we introduce the existing model—extensive games with cognition—in Section 2.
(2) Game solving with adversarial cognition. In Section 4, a new algorithmic procedure for solving extensive games with adversarial cognition is presented, and the resulting solution of this algorithm is defined. Since the new solution concept is obtained based on a player’s reasoning about their opponents’ cognition, the strategy is not guaranteed to be the absolute best: this scenario is the reality that practical players face. A series of issues are discussed regarding the correctness and computational complexity of the game-solving algorithm, the existence of the ACE, and its connection with the CPE.
(3) Examples and reasonability of the model. For a better understanding of this model, Section 6 is devoted to an illustrative example. In addition, this framework is shown to be reasonable for practical game-playing.

## 2. Preliminaries: Cognitive Extensive Games

This section aims to introduce a game framework [26] that characterizes players’ cognition when playing extensive games by incorporating ANNs into the classic model of extensive games.

### 2.1. Game Models

First, we introduce the concept of (finite) extensive games characterized by pure strategies with perfect information.

Extensive form-perfect information games An extensive form-perfect information game [31] is formally defined as a tuple *G* = $\left(N,T,t,\Sigma_{i},\rho_{i}\right)$, in which

- *N* represents the set of ***players***, and N≠∅;
- $T=(V,A,{\left\{\overset{a}{\to }\right\}}_{a\in A})$ denotes the (directed, irreflexive and finite) game tree, which consists of a set of ***nodes*** (or vertices) *V*, a set of moves or ***actions** A*, and a set of ***arcs*****${\left\{\overset{a}{\to }\right\}}_{a\in A}\subseteq V^{2}$**. For any two nodes *v* and $v^{\prime }$, $v^{\prime }$ is said to be an immediate *successor* to *v* if $v\overset{a}{\to }v^{\prime }$. Nodes without successors are called *leaves* (terminal nodes) and are denoted by *Z*. The set of moves available at *v* is denoted as $A_{v}=\left\{a\in A|v\overset{a}{\to }v^{\prime },v^{\prime }\in V\right\}$;
- t:N→V∖Z denotes the ***turn function*** and indicates which player is to move at each nonterminal node;
- The utility function $\rho_{i}:Z\to R$ determines the utility of each terminal node in *Z* for each player i∈N;
- $\Sigma_{i}$ denotes the set of ***strategies*** $\sigma_{i}$ for player *i*. Each $\sigma_{i}:\left\{v\in V\setminus Z|\;t\left(v\right)=i\right\}\to V$ is a function assigned to every nonterminal node *v*, with t(v)=i acting as an immediate successor of *v*.

A ***strategy profile*** $\sigma ={\left(\sigma_{i}\right)}_{i\in N}$ is a combination of the strategies of every player. The set of all strategy profiles is denoted as Σ. For any player i∈N, $\sigma_{-i}$ is the strategy of players in N∖i. An ***outcome*** function O:Σ→Z is a function that assigns a terminal node to each strategy profile. $O(\sigma_{-i})$ denotes the *outcomes* that can be achieved by agent *i*, given that all the other players follow σ, and $O(\sigma_{i}^{\prime },\sigma_{-i})$ depicts the *outcome* when player *i* follows $\sigma_{i}^{\prime }$ and the other players utilize σ.

An alternative way to depict players’ payoff is through preference relation ${⪰}_{i}$, such that for each player *i*, $v{⪰}_{i}v^{\prime }$ if $\rho_{i}\left(v\right)\geq \rho_{i}\left(v^{\prime }\right)$. The indifference case is written as $v{∼}_{i}v^{\prime }$ when $\rho_{i}\left(v\right)=\rho_{i}\left(v^{\prime }\right)$. We focus on games with a *finite horizon* and no infinite branches in the game tree. In this paper, by “extensive games”, we refer to extensive form-perfect information games with a finite horizon.

For an extensive game *G* and any node v∈V, the ***subgame*** of *G* following *v* is defined as ${G|}_{v}$ =$(N{|}_{v},T{|}_{v},t{|}_{v},$$\Sigma_{i}{|}_{v},\rho_{i}{|}_{v})$, in which ${N=N|}_{v}$, ${T|}_{v}$ is the subtree of *T* with rooted *v* and ${Z|}_{v}{=Z\cap V|}_{v}$, ${t|}_{v}=V{{|}_{v}\setminus Z|}_{v}\to N$ satisfies that ${t|}_{v}\left(v^{\prime }\right)=t\left(v^{\prime }\right)$, $\Sigma_{i}{|}_{v}$ is the set of strategies $\sigma_{i}{|}_{v}$, such that for each $v^{\prime }{\in V|}_{v}$ with ${t|}_{v}\left(v^{\prime }\right)=i$, $\sigma_{i}{|}_{v}\left(v^{\prime }\right)=\sigma_{i}\left(v^{\prime }\right)$, and $\rho_{i}{|}_{v}=\rho_{i}\cap \left(V{|}_{v}\right)$. The outcome of σ in subgame ${G|}_{v}$ is written as ${O|}_{v}\left(\sigma {|}_{v}\right)$.

While this approach gives a full picture of the game from an omniscient observer’s point of view, the extensive game lacks consideration of the players’ vision of the game, which might differ from the real game being played. Normally, players view the game according to accumulated experience, including their judgment of the plausibility of future moves and the suitability of game configurations. That is, a player’s view of the game is only a part of the original game tree, which is narrow and short due to their limited abilities.

In [26], a new model of extensive games was proposed by considering players’ cognition of the game. Technically, two kinds of ANNs were introduced into the game model to model and simulate agents’ cognition.

The first type of ANN, called a *filter net*, represents the players’ cognition regarding the plausibility of future moves. For a filter net FN of a game *G*, the input is any state of *G* and the output is a probability function ff:V×A→R over all future moves at that state. For a state *v* and a possible move *a* at *v*, the probability of choosing *a* at *v* is defined as ff(v)(a), which is usually written as $ff_{v}^{a}$.

The second type of ANN, the *evaluation net*, simulates players’ evaluation of the quality of game states. For an evaluation net EN of a game *G*, the input is any state of *G*, and the output is an evaluation function ef:V→R assigning a probability to each state. For any state *v*, ef(v) predicts the probability of winning the game following *v* and is usually simply written as $ef_{v}$.

The cognitive gameplay process can be modeled based on the filter net and evaluation net. For each decision at a current state, four subprocedures are involved: the first three capture players’ cognition by obtaining the filtration $T^{s}$ of the game tree *T* of an extensive *G*.
     **Algorithm for obtaining the filtration $Fil^{s}\left(T\right)$**1.***Branch-Pick.*** According to prior knowledge, the branches of the current decision point can be narrowed by selecting several (e.g., *b*) of the most plausible alternatives among all the available actions. Formally, for any state *s*, the branches $s^{0}$ corresponding to $\arg\max_{b}\left\{ff\left(s\right)\left(a\right)|a\in A_{s}\right\}$ will be chosen; that is, only the top *b* elements of ff(s)(a) are selected.2.***Subtree-Pick.*** To make decisions, the current player searches the subsequent game tree by following these branches. Due to computational limitations, the exploration of the future involves a finite number of steps (e.g., *l*). Each branch rooted at $s^{0}$ is extended to depth *l*, i.e., the subtree that can be reached within *l* steps is obtained.3.***Evidence-Pick.*** To choose the optimal of the *b* branches, the player evaluates the goodness based on the payoffs of the final states of the subtrees. This evaluation of the game state depends on two aspects: (1) an evaluation via prior knowledge and (2) a vague prediction of the future. The former can be given by the evaluation net, while the latter requires a simulation of future moves, which is necessary, since even if it is difficult for players to obtain the complete game tree, they can still hold a vague prediction of the far future. This process is completed in the following manner. First, for each leaf $s^{l}$ in the subtree following $s^{0}$, a path is computed until the end of the game, where at each node along the path, the most optimal action according to the plausibility returned by ff is chosen. Then, the payoff of the final outcome is determined following $s^{l}$ as the simulation result of $s^{l}$. The overall payoff of $s^{l}$ is the weighted sum of the two parts.

The fourth subprocedure is performed based on the filtration $Fil^{s}\left(T\right)$. Given the cognition on the game tree and the suitability of game states, the player who moves at *s* can choose the optimal move from the *b* options via BI.

Therefore, the game being played is different from the ideal model of extensive games: players’ cognition regarding the game must be included. The following is a model called *cognition games*, which was proposed in [26].

Cognition Games. Given an extensive game *G* = $\left(N,T,t,\Sigma_{i},\rho_{i}\right)$, a filter net FN and an evaluation net EN for *G*, a cognition game $G^{s}$ for *G* at any state *s* is a tuple $\left(N^{s},\;T^{s},\;t^{s},\;\Sigma_{i}^{s},\;\rho_{i}^{s}\right)$, in which

- $T^{s}$ denotes the filtration $Fil^{s}\left(T\right)$ of *T* at *s*;
- The set of players is $N^{s}=\left\{i\;|\;t\left(s^{\prime }\right)=i\right\}$ for $s^{\prime }\in T^{s}$;
- Turn function $t^{s}$ is consistent with *t*, i.e., $t^{s}\left(s^{\prime }\right)=t\left(s^{\prime }\right)$ for any $s^{\prime }$;
- The set of strategies $\Sigma_{i}^{s}$ coincides with $\Sigma_{i}$. $\sigma_{i}^{s}\left(s^{\prime }\right)=\sigma_{i}\left(s^{\prime }\right)$ for every $s^{\prime }$ with $t(s^{\prime })=i$;
- The utility function $\rho_{i}^{s}$ is an integration of the results obtained via the evaluation net and simulation. Let $z^{s}$ be any leaf of $T^{s}$, and let *z* be the final point following $z^{s}$ in the simulation process. Then, $\rho_{i}^{s}\left(z^{s}\right)=\left(ef\left(z^{s}\right)+\rho_{i}\left(z\right)\right)/2$.

### 2.2. Equilibrium Concepts

A substantial concern in games is the equilibrium concept, which characterizes a state of dynamic balance in which no player can deviate from this strategy and improve their expected payoff. Two classic equilibrium concepts for extensive games are the Nash equilibrium [32] and SPE.

*Nash equilibrium*. Let $\sigma^{*}$ be any strategy profile of an extensive game *G*. $\sigma^{*}$ is the *best response* for player *i* if $O\left(\sigma_{i}^{*},\sigma_{-i}^{*}\right){⪰}_{i}O\left(\sigma_{i},\sigma_{-i}^{*}\right)$ for each strategy $\sigma_{i}$ of *i*. If $\sigma^{*}$ is the *best response* for all players *i*, then $\sigma^{*}$ is called a *Nash equilibrium* of *G*.

*Subgame perfect equilibrium*. Let $\sigma^{*}$ be any strategy profile of an extensive game *G*. If, for each player, *i* and any node *v* with t(v) = *i*, ${O|}_{v}(\sigma_{i}^{*}{|}_{v},\sigma_{-i}^{*}{|}_{v}{)\;{⪰}_{i}\;O|}_{v}\left(\sigma_{i},\sigma_{-i}^{*}{|}_{v}\right)$ holds for any of *i*’s strategies $\sigma_{i}$ in ${G|}_{v}$, and $\sigma^{*}$ is called an SPE of *G*.

The *best response* is the best option for each player given what other players will do. Consequently, the *Nash equilibrium* requires that a strategy profile consists of the best response for every player. SPE considers sequential moves and must be the Nash equilibrium in all subgames.

For any extensive game *G* with root $v_{0}$, an SPE σ of *G* can determine a node sequence $\left(v_{0},v_{1},\cdots ,v_{k}\right)$, s.t. for each 0≤i≤(k−1), $\sigma \left(v_{i}\right)=v_{i+1}$, and $v_{k}$ is a terminal node. We call this node sequence a σ-SPE solution of *G*, which is written as $q_{\sigma }$.

A fundamental way to find the SPE of extensive games is BI [33], which identifies the best move for the player who is the last to move in the game. Subsequently, the optimal move for the next-to-last player is then found. This process is repeated from the end of the game to the beginning to determine the strategies for all players.

An apparent weakness of BI is the need to search the full game tree, which makes the approach impractical for large-scale games. Due to resource limitations or ability constraints [34], players cannot make a perfect prediction of the future in practical game-playing processes. Normally, players must make decisions via a heuristic search over a limited part of the game tree based on prior knowledge. By taking players’ cognition about the game into consideration, cognitive game-solving provides a more realistic framework for playing extensive games. The resulting equilibrium concept is called CPE.

Cognitive Perfect Equilibrium [26]. For an extensive game *G* = $\left(N,T,t,\Sigma_{i},\rho_{i}\right)$, a filter net FN and an evaluation net EN of *G*, a strategy profile $\sigma^{*}$ is called a CPE of *G* if and only if the following holds:

For each v∈V∖Z and each node $u\in G^{v}$ ($t^{v}\left(u\right)=i$), there is a strategy profile $\sigma^{v}$ of $G^{v}$ satisfying $\sigma^{*}\left(v\right)=\sigma^{v}\left(v\right)$ and $O_{i}^{v}\left(\sigma^{v}{|}_{u}\right)$${⪰}_{i}$$O_{i}^{v}(\sigma_{-i}^{v}{|}_{u},\sigma_{i}^{\prime }{|}_{u})$ for any strategy profile $\sigma^{\prime }$ of *i* in $G^{v}$.

The intuition of CPE is that at every decision point, the CPE is consistent with the SPE of the corresponding cognition game.

Cognitive games provide realistic representations of extensive games, and the CPE reflects the gameplay procedure. However, a major drawback exists concerning cognitive games and the CPE. In cognitive games, players assume that their cognition is consistent with that of their opponents, i.e., their opponents have the same view of the game being played and the same evaluation of the game states.

As a result, cognitive games omit the player’s reasoning on their opponents’ cognition, which might play significant roles in the player’s strategies. In particular, the player may benefit from exploiting the opponents’ cognition.

This paper aims to refine cognitive games by endowing players with the ability to learn their opponents’ cognition about the game being played and to evaluate game situations. An appropriate solution concept is obtained under this new game model.

## 3. Adversarial Cognition Game

In this section, we introduce a refinement of cognition games, in which players are allowed to learn and reason about the cognition of their opponents, namely, the game the opponents believe is being played and their evaluation of the game situations, i.e., the utility functions they use. We first introduce the notion of *state pair*, a formal structure that allows reasoning about the cognition of opponents.

***State Pairs***. Consider an extensive game *G* = $\left(N,T,t,\Sigma_{i},\rho_{i}\right)$, a filter net FN, and an evaluation net EN for *G*. A *state pair* π of *G* is a pair of states of the form $\left(v_{0},v_{1}\right)$ satisfying $v_{1}\in T^{v_{0}}$, i.e., states following $v_{0}$ in the pair are those within the filtration at $v_{0}$.

Without loss of generality, we assume that the opponents’ cognitive ability is encoded by the number of future steps that are foreseeable to them, i.e., their search depth. The opponents’ evaluation of the goodness of leaves of the search tree can be modeled as a static payoff function *p*. The set of utility functions is denoted by *P*. The intuition behind *state pairs* is to capture the adversarial cognition of the player who moves at $v_{0}$. The expression of the form $\left(v_{0},v_{1}\right)$ encodes the cognition that the player moving at $v_{0}$ holds about the cognition of the player moving at $v_{1}$, including what he can foresee occurring in the future and what his utility function is. We use Π to denote the set of state pairs of *G*.

Based on the notion of state pairs, we can represent *adversarial cognition structures by* associating each state pair with a set of states and an evaluation over the terminal states therein.

***Adversarial cognition structures.*** Let *G* be an extensive game and FN and EN be the filter net and evaluation net for *G*, respectively. An *adversarial cognition structure* C for *G* is a tuple $\left(C_{V},C_{E}\right)$, such that $C_{V}$ is a function $C_{V}:\Pi \to 2^{V}$, associating a subset of nodes following $v_{1}$ with each state pair $\left(v_{0},v_{1}\right)$, and $C_{E}$ is a function $C_{E}:\Pi \to P$, associating a payoff function with each state pair. C satisfies the following conditions:

- (Self-consistence) ∀π∈Π with $\pi =(v_{0},v_{1})$, we have that $C_{V}\left(v_{0},v_{1}\right)=V^{v_{1}}$ whenever $t\left(v_{1}\right)=t\left(v_{0}\right)$, i.e., the player has a precise cognition regarding what he himself can foresee.
- (Adversary–inclusivity) ∀$\pi =\left(v,v^{\prime }\right),\pi^{\prime }=\left(v,v\right)\in \Pi$, $C_{V}\left(\pi \right)\subseteq C_{V}\left(\pi^{\prime }\right)$, i.e., an agent’s cognition for $\left(v,v^{\prime }\right)$, is a subset of his cognition of himself at (v,v).
- (Depth-limited) ∀π∈Π with $\pi =(v_{0},v_{1})$ and $t\left(v_{1}\right)\neq t\left(v_{0}\right)$, we have that $C_{V}\left(v_{0},v_{1}\right)$ = $V^{v_{0}}{|}_{v_{1}}^{d}$, where d∈N, i.e., the player’s cognition at $v_{0}$ about what the player moving at $v_{1}$ can see is represented by nodes in the subtree limited by a depth *d* (we use $V^{v_{0}}{|}_{v_{1}}^{d}$ to denote the nodes in $V^{v_{0}}{|}_{v_{1}}$ that can be reached from $v_{1}$ within *d* depth).

For $\pi =(v_{0},v_{1})$, $C_{E}\left(\pi \right)$ denotes the player’s cognition regarding the utility function of the player moving at $v_{1}$.

A game model with the agent’s cognition regarding their opponents can then be obtained by assigning an adversarial cognition structure to a cognition game.

***Adversarial Cognition Game.*** An *adversarial cognition game* (ACG) is defined as a tuple Γ=(G,FN,EN,C), with *G* being an extensive game, while FN, EN and *C* are the filter net, evaluation net, and adversarial cognition structure for *G*, respectively.

Note that an ACG induces a sequence of extensive games, one for each state pair concerning the player’s adversarial cognition. For any ACG Γ, we denote the game induced by any sate pair π as $\Gamma_{\pi }$, for which the game tree is restricted by $C_{V}\left(\pi \right)$ and the utility function is $C_{E}\left(\pi \right)$.

## 4. Game Solving and Equilibrium

**Figure 1:**
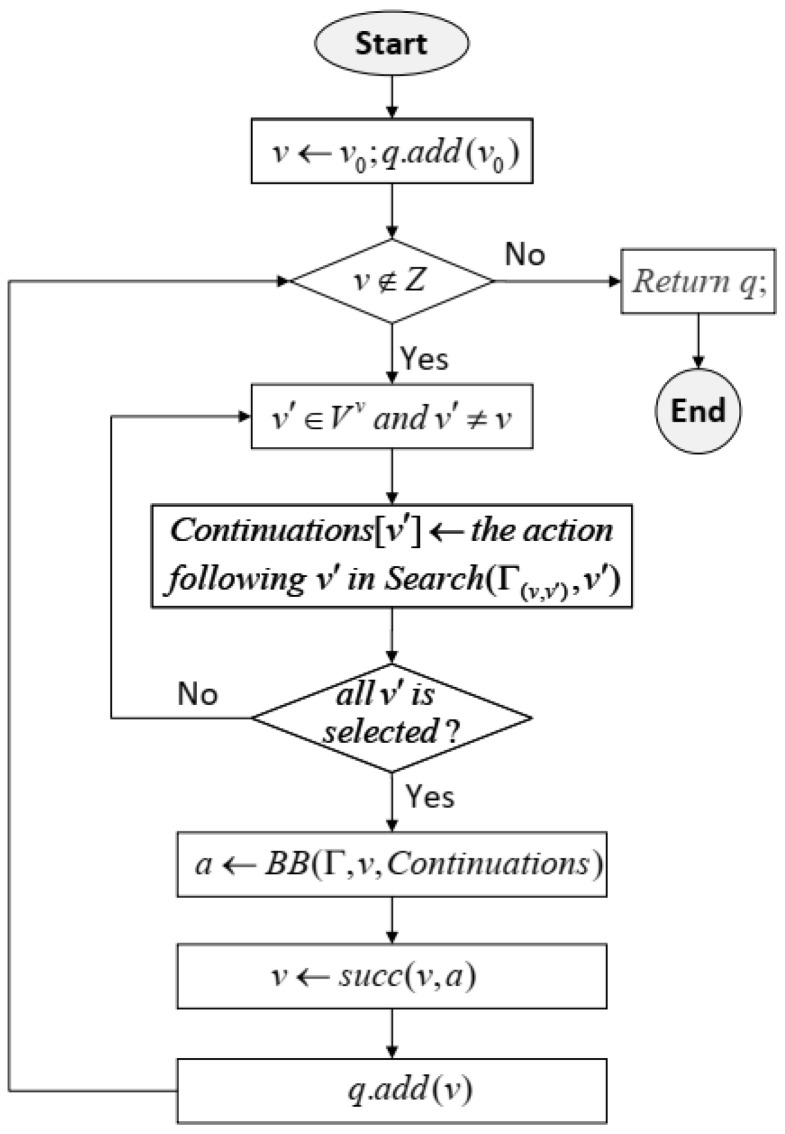
Flow chart of adversarial cognitive game solving.

Based on the player’s adversarial cognition at each state pair, he can search the current game and make an optimal decision with regard to the possible moves and payoffs for the corresponding outcomes. The combination of all these optimal decisions results in a solution to the ACG. According to this idea, Algorithm 1 presents the process for solving an ACG, which is called *adversarial cognitive game solving*. The process starts from root $v_{0}$ and extends the sequence *q* by successively adding the successor node that is the result of the optimal move in the cognition game at the current node determined by using Algorithm 2. This process is depicted in Figure 1.
**Algorithm 1:** Solution of Γ.
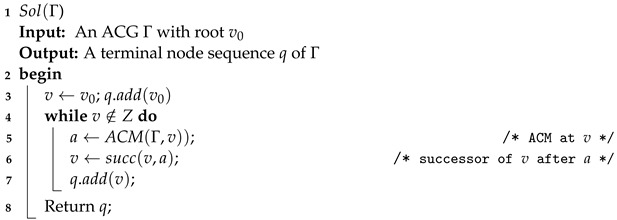

**Algorithm 2:** Adversarial cognition move.
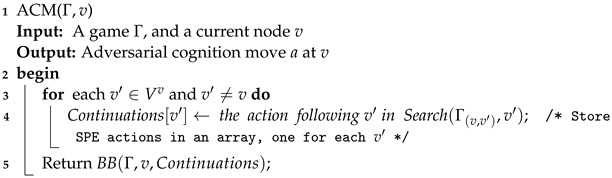


The most important parts of Algorithm 2 are the function Search (Algorithm 3) and function BB (Algorithm 4). Algorithm 3 computes, for a state pair $\pi =(v,v^{\prime })$, the node sequences determined by the SPE of $\Gamma_{\pi }$ and then yields the optimal successors following *v*. Given the optimal move of each node $v^{\prime }$ in the cognition game $G^{v}$ of the current player at *v*, Algorithm 4 computes a best path following *v* for t(v) and returns the immediate successor of *v* as required.
**Algorithm 3:** Search.
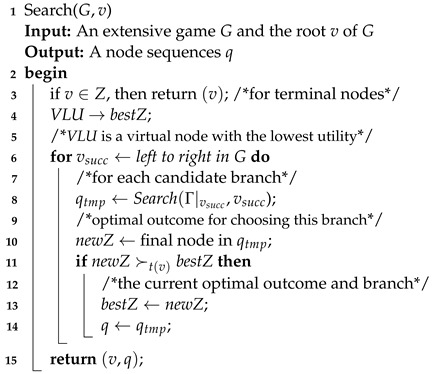

**Algorithm 4:** Best Branch.
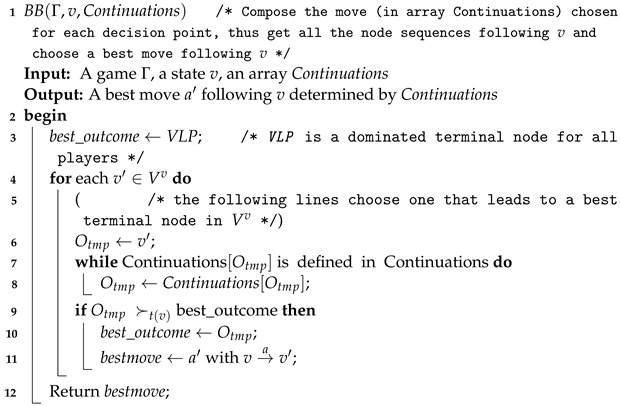


Note that the above game-solving algorithm is different from the standard BI or the cognitive game-playing process in [26]. Consequently, the resulting equilibrium of the ACG differs from that of the SPE or CPE. Algorithm 3 searches the game $\Gamma_{\left(v,v^{\prime }\right)}$ induced by state pair $\left(v,v^{\prime }\right)$, which corresponds to an SPE of $\Gamma_{\left(v,v^{\prime }\right)}$. The optimal move at state *v* should be consistent with the SPE of $\Gamma_{\left(v,v^{\prime }\right)}$ for any state $v^{\prime }$ within t(v)’s cognition game $G^{v}$. Therefore, the first equilibrium we can define is the one obtained at state *v*, which is called *local adversarial cognition equilibrium* (LACE).

***LACE.*** Let Γ=(G,FN,EN,C) be an ACG, and let *v* be any node in *V*. A strategy profile $\sigma^{v}$ is a LACE of Γ at *v* if:

- At each $v^{\prime }\in G^{v}$, there exists a terminal node $z\in Z_{\left(v,v^{\prime }\right)}$ in $\Gamma_{\left(v,v^{\prime }\right)}$, such that $\sigma^{v}\left(v^{\prime }\right)⊴z$ and *z* is an outcome of the SPE for $\Gamma_{\left(v,v^{\prime }\right)}$.

We denote LACS(Γ,v) as the set of LACE outcomes of Γ at *v*. The composition of such outcomes yields the global solution for Γ:

***Adversarial Cognition Equilibrium.*** Let Γ=(G,FN,EN,C) be an ACG. A strategy profile σ is an *adversarial cognition equilibrium* (ACE) of Γ if, at each v∈V∖Z, there exists a terminal history $z\in Z^{v}$ in $G^{v}$, such that σ(v)⊴z and z∈LACS(Γ,v).

An ACS is the composition of best strategies of players at each decision node. Each such strategy is the best response for the players, given the player’s cognition about the opponents’ beliefs on the games being played and the quality of the game states.

As is the case for other equilibrium concepts, each ACE of an ACG also determines a specific sequence of nodes. Suppose σ is an ACE of Γ with root $r_{0}$. The **ACE solution determined by σ**, dubbed as $q_{\sigma }$, is a sequence of $\left(v_{0},v_{1},\cdots ,v_{k}\right)$, satisfying that $v_{0}=r_{0}$, $v_{k}\in Z$, and $\sigma \left(v_{i}\right)=v_{i+1}$ for all *i* with 0≤i≤k−1.

The set of adversarial cognition solutions of Γ is denoted as ACS.

For game theory, another fundamental concern is the *existence* of an equilibrium. The following lemma clarifies that every ACG has an ACE.

**Lemma 1.**  *(Existence) Every ACG Γ has an ACE.*

**Proof.**  It is sufficient to show the existence of the ACE at any position *v*. The first step is to prove the existence of the SPE for each $\Gamma_{\left(v,v^{\prime }\right)}$. We can obtain an SPE at *v* via induction with regard to the depth *h* of the nodes. Let *f* be a function that connects a path with each state $v^{\prime \prime }\in \Gamma_{\left(v,v^{\prime }\right)}$. When h=0, i.e., $v^{\prime \prime }$ is a leaf, define $f\left(v^{\prime \prime }\right)=\left(v^{\prime \prime }\right)$. Then, if $f(v^{\prime \prime })$ is defined for all nodes with a depth of h≤k for some k>0, suppose $v^{*}$ is a node with $h(v^{*})=k+1$, and $t(v^{*})=i$. Given that $h(v^{*})=k+1$, it has $h(u^{*})\leq k$, where $v^{*}\overset{a}{\to }u^{*}$ for any action *a* in $\Gamma_{\left(v,v^{\prime }\right)}$. Let $\sigma_{i}\left(v^{*}\right)$ be a maximizer of $f(v^{*})$ over followers of *v* in $\Gamma_{\left(v,v^{\prime }\right)}$, and let $f\left(v^{*}\right)=f\left(\sigma_{i}\left(v^{*}\right)\right)$. Via an induction, we have obtained a strategy profile σ in $\Gamma_{\left(v,v^{\prime }\right)}$. According to the definition of SPE, σ is an SPE of $\Gamma_{\left(v,v^{\prime }\right)}$.

For any intermediate node *v* in Γ, let $\sigma^{\prime }\left(v^{\prime }\right)=\sigma \left(v^{\prime }\right)$ for every $v^{\prime }$ in $G^{v}$, in which σ is an SPE of $\Gamma_{\left(v,v^{\prime }\right)}$. According to the definition, $\sigma^{\prime }$ is an ACE of Γ at *v*. Finally, we can construct a strategy $\sigma^{*}=\sigma^{\prime }\left(v\right)$ for every state *v* in Γ; thus, it is evident that $\sigma^{*}$ is an ACE of Γ. □

The observation below illustrates the connection between the ACE and the two previously mentioned equilibrium concepts, SPE and CPE, by specifying the conditions under which the ACE collapses into the SPE or CPE.

**Proposition 1.**  *Let Γ=(G,FN,EN,C) be an ACG.*

*(1)*  *If for every nonterminal node v, $\Gamma_{\left(v,v^{\prime }\right)}$ = $G^{v}{|}_{v^{\prime }}$ for any node $v^{\prime }$ in the filtration $G^{v}$ at v, then an ACE of Γ is also a CPE, and vice versa.*
*(2)*  *If for every nonterminal node v, $G^{v}{=G|}_{v}$ and $\Gamma_{\left(v,v^{\prime }\right)}$ = ${G|}_{v^{\prime }}$ for any node $v^{\prime }$ in the filtration $G^{v}$ at v, then an ACE of Γ is also an SPE of G, and vice versa.*

**Proof.**  (1)(⇐). Let $\sigma^{*}$ be a CPE of Γ. For all the nonterminal nodes *v* and any node $v^{\prime }$ in $G^{v}$ with $t^{v}\left(v^{\prime }\right)=i$, there is a $\sigma^{v}$ with $O^{v}\left(\sigma^{v}{|}_{v^{\prime }}\right)$${⪰}_{i}$$O^{v}(\sigma_{-i}^{v}{|}_{v^{\prime }},\sigma_{i}^{\prime }{|}_{v^{\prime }})$ for any alternative $\sigma^{\prime }$ in $G^{v}$, satisfying that $\sigma^{*}\left(v\right)=\sigma^{v}\left(v\right)$. When $\Gamma_{\left(v,v^{\prime }\right)}$ = $G^{v}{|}_{v^{\prime }}$, we find that $\sigma^{v}$ is an SPE of $\Gamma_{\left(v,v^{\prime }\right)}$ for any $v^{\prime }$ in $G^{v}$. Therefore, $\sigma^{v}$ is a LACE of Γ at each *v*. Since $\sigma^{*}\left(v\right)=\sigma^{v}\left(v\right)$ for any *v*, $\sigma^{*}$ is an ACE of Γ by definition. (⇒) Let $\sigma^{*}$ be an ACE of Γ. For all the nonterminal nodes *v*, there is a $\sigma^{v}$ that is a LACE of Γ with $\sigma^{*}\left(v\right)=\sigma^{v}\left(v\right)$. That is, for any $v^{\prime }$ in $G^{v}$, there is an SPE $\sigma^{\prime }$ of $\Gamma_{\left(v,v^{\prime }\right)}$, such that $\sigma^{v}\left(v^{\prime }\right)$ = $\sigma^{\prime }\left(v^{\prime }\right)$. When $\Gamma_{\left(v,v^{\prime }\right)}$ = $G^{v}{|}_{v^{\prime }}$, it holds that $\sigma^{\prime }$ is an SPE of $G^{v}{|}_{v^{\prime }}$ for any such $v^{\prime }$. Therefore, $\sigma^{v}$ is an SPE of $G^{v}$ for any *v*. Since $\sigma^{*}\left(v\right)=\sigma^{v}\left(v\right)$, it follows that $\sigma^{*}$ is a CPE of Γ.

(2)(⇐). Let $\sigma^{*}$ be an SPE of *G*. For every nonterminal node *v* with t(v) = *i*, ${O|}_{v}(\sigma_{i}^{*}{|}_{v},\sigma_{-i}^{*}{|}_{v}{)\;{⪰}_{i}\;O|}_{v}\left(\sigma_{i},\sigma_{-i}^{*}{|}_{v}\right)$ holds. Consequently, for any $v^{\prime }{\in G|}_{v}$, ${O|}_{v^{\prime }}(\sigma_{i}^{*}{|}_{v^{\prime }},\sigma_{-i}^{*}{|}_{v^{\prime }})$${⪰}_{t(v^{\prime })}{\;O|}_{v^{\prime }}\left(\sigma_{i},\sigma_{-i}^{*}{|}_{v^{\prime }}\right)$ holds. Given that $G^{v}{=G|}_{v}$ and $\Gamma_{\left(v,v^{\prime }\right)}$ = ${G|}_{v^{\prime }}$, there is a strategy $\sigma^{v}$ with $O^{v}\left(\sigma^{v}{|}_{v^{\prime }}\right)$${⪰}_{i}$$O^{v}(\sigma_{-i}^{v}{|}_{v^{\prime }},\sigma_{i}^{\prime }{|}_{v^{\prime }})$ for any alternative $\sigma^{\prime }$ in $G^{v}$, satisfying that $\sigma^{*}\left(v\right)=\sigma^{v}\left(v\right)$. That is, $\sigma^{v}$ is a LACE of Γ at each *v*; thus, $\sigma^{*}$ is an ACE of Γ. (⇒). Take any ACE $\sigma^{*}$ of Γ. For any nonterminal node *v*, there exists a strategy $\sigma^{v}$, which is a LACE of Γ. That is, for any $v^{\prime }$ in $G^{v}$, there is an SPE $\sigma^{\prime }$ of $\Gamma_{\left(v,v^{\prime }\right)}$, s.t., $\sigma^{v}\left(v^{\prime }\right)$ = $\sigma^{\prime }\left(v^{\prime }\right)$. If $G^{v}{=G|}_{v}$ and $\Gamma_{\left(v,v^{\prime }\right)}$ = ${G|}_{v^{\prime }}$, we find that $\sigma^{\prime }$ is an SPE of ${G|}_{v^{\prime }}$ for any such $v^{\prime }$. Therefore, $\sigma^{v}$ is an SPE of ${G|}_{v}$ for any *v*. Since $\sigma^{*}\left(v\right)=\sigma^{v}\left(v\right)$, it follows that $\sigma^{*}$ is an SPE of *G*. □

Therefore, if the current player’s cognition regarding the following players’ cognition is the same as his cognition of himself, then the ACE is equivalent to the CPE; if the player’s cognition is the same as the complete subtree therein, then the ACE is equivalent to the SPE. However, these conditions are normally impossible during real gameplay, which reflects the rationality of our framework.

Crucial issues concerning the game-solving algorithm include its correctness and complexity. The following theorem presents an argument that each solution returned by Algorithm 1 is an ACS of the game.

**Theorem 1.**  (Correctness) For any ACG Γ with root $r_{0}$, for any path $q^{*}$ returned by Sol(Γ) in Algorithm 1, there exists an ACE $\sigma^{*}$ of Γ, such that $q_{\sigma^{*}}=q^{*}$.

**Proof.**  This can be proved by induction regarding the depth *d* of the game tree.

Base case: d=1 is trivial, with only a single node $r_{0}$ in the game.

When d=2, let $q^{*}=\left(r_{0},z_{1}\right)$. According to Algorithm 1, $\left(z_{1}\right)$ is a successor of *v* obtained by executing $ACM(\Gamma ,r_{0})$ (Lines 5–7). That is, $\left(z_{1}\right)$ is a sequence returned by $Search(\Gamma_{\left(r_{0},z_{1}\right)},z_{1})$ (line 4 in Algorithm 2) and the action *a*, such that $r_{0}\overset{a}{\to }z_{1}$ is a best move returned by $BB(\Gamma ,r_{0},Continuations)$. Therefore, $\left(z_{1}\right)$ is an SPE solution of $\Gamma_{(}r_{0},z_{1})$. At the same time, $\left(z_{1}\right)$ is a LACS of Γ at $r_{0}$. We can define a strategy profile $\sigma^{*}$ as $\sigma^{*}\left(r_{0}\right)=z_{1}$, and $\sigma^{*}\left(z\right)=z$ for any $z\in Z^{r_{0}}$. Observe that $\sigma^{*}$ is an ACE of Γ according to the definition of ACE. Hence, $q^{*}$ is determined by an ACE.

Induction assumption: For Γ with depth *k*, $q^{*}=q_{\sigma^{*}}$.

Induction: Let $q^{*}=\left(r_{0},v_{1},\cdots ,v_{k}\right)$ be a node sequence returned by Algorithm 1. Therefore, $\left(v_{i+1}\right)$ initiates an SPE solution of $\Gamma_{\left(r_{0},v_{i}\right)}$. We can define a strategy profile $\sigma^{*}$ as $\sigma^{*}\left(r_{0}\right)=v_{1}$ and $\sigma^{*}\left(v_{i}\right)=\sigma \left(v_{i}\right)$ for $v_{i}\in \left\{v_{1},\cdots ,v_{k}\right\}$, and for any other state $v^{\prime }$, $\sigma^{*}\left(v^{\prime }\right)$ is consistent with an SPE of $\Gamma_{\left(r_{0},v^{\prime }\right)}$. Then, $q^{*}=q_{\sigma^{*}}$. It has yet to be verified that $\sigma^{*}$ is an ACE of Γ. This result is direct according to the definition of ACE. □

The complexity of Algorithm 1 is analyzed in the following proposition.

**Proposition 2.**  *(Complexity) The worst time complexity of Algorithm 1 is $O(n\log^{2}n)$, where n is the number of nodes in the underlying game.*

**Proof.**  First, let *b* be the number of branches selected in the filtration and let *d* be the depth of the game; then, the time complexity of Algorithm 4 is T(BB) = O(b∗d). For Algorithm 3, let t(d) be the complexity of a game tree with depth *d*. Then, t(d)=O(b∗t(d−1)), and for any *k* = 1,⋯,l, t(d−k)=O(m∗t(d−k−1)), where *m* represents the width of the game tree. Meanwhile, t(d−l−j)=1 for j=1,⋯,(d−2). Through iterative computation, the time complexity of Algorithm 3 is obtained, i.e., $t\left(d\right)=O\left(b*m^{l}\right)$. Algorithm 2 must first obtain the filtration at *v*, then call Algorithm 3 for each $v^{\prime }$ in the filtration, and finally call Algorithm 4. Therefore, the time complexity is the sum of the three parts. For the filtration, the complexities of the three subprocedures are $f_{1}\left(G^{v}\right)=m*b$, $f_{2}\left(G^{v}\right)=O\left(b*\left(m+m^{2}+\cdots +m^{\left(l-1\right)}\right)\right)=O\left(b*m^{l}\right)$ and $f_{3}\left(G^{v}\right)=O\left(b*m^{l-1}*\left(d-l-1\right)*m\right)$. Considering that *b* and *l* are normally much smaller than *m* and *d*, we can obtain the complexity of the filtration at *v*, i.e., $f\left(G^{v}\right)=O\left(m*b+b*m^{l}+b*m^{l-1}*\left(d-l-1\right)*m\right)=O\left(d*m^{l}\right)$. Therefore, the complexity of Algorithm 2 is $T\left(ACM\right)=f\left(G^{v}\right)+O\left(b*d\right)*t\left(d\right)+T\left(BB\right)=O\left(b^{2}*d*m^{l}\right)$. To obtain a node sequence *q*, the ACM musts be called O(d) times. Hence, the overall complexity of Algorithm 1 is $T=O\left(b^{2}d^{2}m^{l}\right)=O\left(n\log^{2}n\right)$, where *b* is a constant and $m^{d}=O\left(n\right)$, l≪d, d=O(logn). □

## 5. An Example: Tic-Tac-Toe

After establishing a model of extensive games involving players’ cognition on the opponents and the new solution concept, we proceed with an example illustrating this framework, through which the procedure of solving such games is demonstrated. Comparison with the case without cognition about opponents confirms the feasibility of opponent modeling in gameplay.

We consider the example first presented in [26], which starts with a scenario in a Tic-Tac-Toe game. Tic-Tac-Toe is a simple game that is suitable for illustrating our model, and has been extensively used in the literature due to its simplicity. According to [26], with a player’s own cognition regarding the game, a cognition game model of Tic-Tac-Toe consists of three components: a classic extensive game model *G*, a filter net FN and an evaluation net EN, where:

(1) $G=(N,T,t,\Sigma_{i},u_{i})$, such that
  - the set of players N={1,2}, with 1for× and 2for∘;
  - game tree *T*=$\left(V,A,{\left\{\overset{a}{\to }\right\}}_{a\in A}\right)$, with
    *V*={legallayoutsofthe3×3board};
    *A*={legalactionsbythegamerule};
    ${\left\{\overset{a}{\to }\right\}}_{a\in A}$ = $\left\{\left(v_{1},v_{2}\right)|v_{1}\overset{a}{\to }v_{2},v_{1}\in V,v_{2}\in V,a\in A\right\}$;
  - t(v)=1 for nodes in which it is player 1’s turn to move, and t(v)=2 for player 2’s turn;
  - player *i*’s strategies $\Sigma_{i}$ = $\left\{\sigma_{i}\right\}$, and $\sigma_{i}\left(v\right)\in A_{v}$ for each $\sigma_{i}$ and v∈V∖Z with t(v)=i;
  - utility $\rho_{i}\left(z\right)$ is defined as 1 for any terminal node *z* where *i* wins the game; $\rho_{i}\left(z\right)=0$ when player *i* loses at *z*; and $\rho_{i}\left(z\right)=0.5$ when there is a draw.
(2) FN is a multi-layer backpropagation (BP) neural network, in which the number of input neurons is nine, representing the feature of nine grids. There are also 9 output neurons, one for each grid (−1 for ×, 1 for ∘, and 0 for idle). There are also 50 hidden neurons. The filter function ff is determined by the output probability p(s,a) of the filter net for any state *s* and any possible move following *s*.
(3) EN shares the same structure as FN, but it has only one output neuron, which outputs a probability p(s) for *s*.

The process for game solving in [26] under this model is to compute the CPE. The decisions at each point are made based on the two output probabilities from the filter net and evaluation net, which characterize players’ cognition on the plausibility of moves and the quality of game states, respectively.

**Figure 2:**
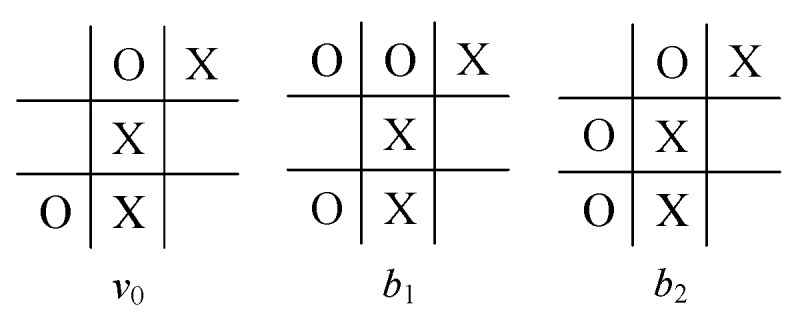
An instance of Tic-Tac-Toe.

**Figure 3:**
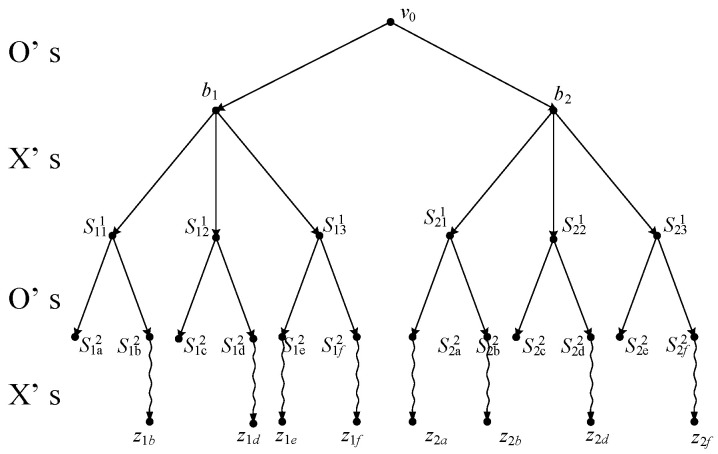
Filtration of the game tree.

**Figure 4:**
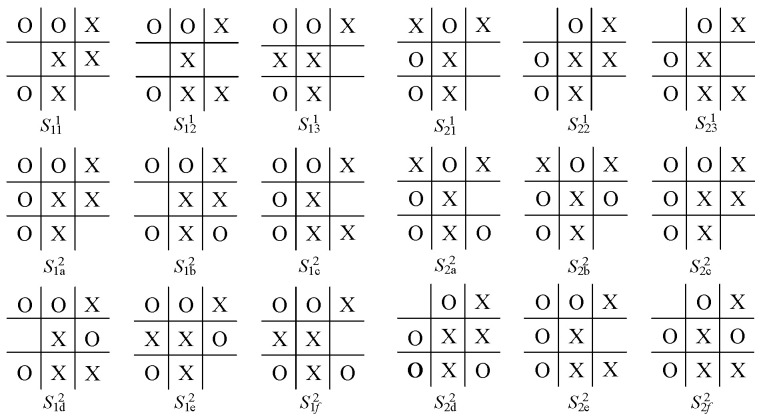
Board configuration of intermediate nodes.

**Figure 5:**
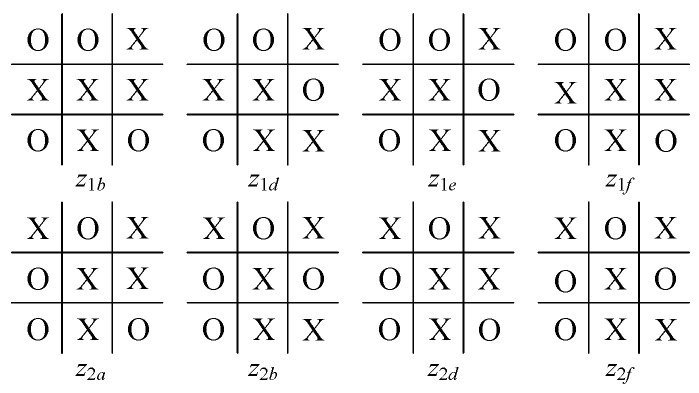
Board configuration of terminal nodes.

For comparison with the model proposed here, we consider the same instance, viz., a partial game of Tic-Tac-Toe, with a starting point $v_{0}$ (see Figure 2 and two of its successors $b_{1}$, $b_{2}$) in which it is player *O*’s turn to move. The game tree after filtration via the filter net FN is shown in Figure 3, where the board configurations of these nodes are shown in Figure 4 (for intermediate nodes) and Figure 5 (for terminal nodes).

The final utilities of the terminal nodes (based on the cognition of player *O*) are shown in Table 1. Note that these nodes are not terminal nodes of the original game, but the terminal ones within the cognition of *O*. For each node, each utility is given as the average of the probability returned by the evaluation net EN and the utility of the leaf in the most plausible subsequent path. For each pair of utilities, the first value is the utility of player *X*, and the second is the utility of player *O*. For simplicity, more details about obtaining the above figures and table are omitted, since the information does not affect our consideration.

According to the cognition game solution algorithm in [26], for the subtree at $b_{1}$, player *X* will choose branch $s_{13}^{1}$ via a BI process, since when using $s^{13}$, *X* can receive a utility 0.30 after *O*’s choice of $s_{1e}^{2}$; similarly, for the subtree at $b_{2}$, the player chooses $s_{21}^{1}$. Consequently, the optimal choice for *O* at $b_{0}$ is $b_{1}$. In the following steps, since there are at most three branches and the search depth is no greater than three, no filtration is needed. Continuing this game-solving procedure results in a leaf node $z_{1d}$, in which the game ends with a draw.

The above process is based on the assumption that player *X* holds the same cognition as player *O*. However, if the player’s cognition on the opponent is involved, the results are different. If the player can make a precise prediction about their opponent, then he can try to utilize this information and obtain a better outcome.

Suppose now that player *X*’s cognition on the game tree is limited to a subtree of depth 2, i.e., the player can only search two steps forward. Moreover, the player’s evaluation function regarding the quality of nodes is given by $f_{X}\left(v\right)=\sum_{j}w\left(j\right)*c\left(j\right)$, where j=1⋯9 denotes the 9 grids on the board, and w(j) and c(j) are the weight and the value for each grid. c(j)=1 if the *j*th grid is occupied by player *X*; c(j)=0 if the grid is blank; otherwise, c(j)=−1. w(j)=3 for four corners; w(j)=2 for the center grid; otherwise, w(j)=1. For example, the evaluation of $s_{1a}^{2}$ (for which c(3)=c(5)=c(6)=c(8)=1, c(1)=c(2)=c(4)=c(7)=−1, c(9)=0; w(1)=w(3)=w(7)=w(9)=3; w(5)=2; w(2)=w(4)=w(6)=w(8)=1, i.e., there is one grid with weight 3 and value 1, one grid with weight 2 and value 1, two grids with weight 1 and value 1, and two grids with weight 3 and value −1, two grids with weight 1 and value −1), one with weight 3 and value 0 is as follows:

$f_{X}\left(s_{1a}^{2}\right)$ = 1∗3+1∗2+2∗1+(−1)∗3∗2+(−1)∗1∗2=−1. Correspondingly, the evaluation of $s_{1a}^{2}$ for player *O* (from the perspective of *X*) is 1. We write this result as $f\left(s_{1a}^{2}\right)=\left(-1,1\right)$.

We can then directly obtain the evaluation for the other nodes following $b_{1}$: $f\left(s_{1b}^{2}\right)=\left(-1,1\right)$; $f\left(s_{1c}^{2}\right)=\left(1,-1\right)$; $f\left(s_{1d}^{2}\right)=\left(1,-1\right)$; $f\left(s_{1e}^{2}\right)=\left(-1,1\right)$; $f\left(s_{1f}^{2}\right)=\left(-3,3\right)$. Similarly, $f(s_{2a}^{2})$ = (1,−1); $f\left(s_{2b}^{2}\right)=\left(3,-3\right)$; $f\left(s_{2c}^{2}\right)=\left(-1,1\right)$; $f\left(s_{2d}^{2}\right)=\left(-1,1\right)$; $f\left(s_{2e}^{2}\right)=\left(1,-1\right)$; $f\left(s_{2f}^{2}\right)=\left(3,-3\right)$.

Furthermore, suppose that player *O*’s prediction on *X*’s cognition is correct. Then, from *O*’s perspective, *X* will choose $s_{12}^{1}$ at $b_{1}$ and $s_{21}^{1}$ or $s_{23}^{1}$ at $b_{2}$. Therefore, player *O* will choose $b_{1}$ at $v_{0}$, since this selection will result in $s_{1c}^{2}$, where *O* wins the game. Compared with the case without reasoning about player *X*’s cognition, player *O* gains a better result (a win or a draw) by knowing the decision that will be made by *X*.

## 6. Conclusions and Future Work

As an important tool for decision analysis in many fields, such as sensors [35,36] and autonomous driving [37], Game Theory needs to be extended to develop more realistic models and equilibrium concepts. Constrained by computing resources, game rules, and other factors, game decision-makers often find it difficult to obtain a complete understanding of their opponents during the decision-making process, and can only make certain speculations about the opponents based on their information. The player’s perception of their opponents greatly affects the quality of their decision-making. Considering the importance of improving the game-playing outcome by utilizing the opponent’s cognition regarding the underlying game, this paper proposed a new model of extensive games, based on which a new equilibrium concept –ACE– was derived. An algorithmic procedure of adversarial cognitive game playing and the learning of opponents’ cognition were also presented. The proposed model and solution concept are shown to be superior to the standard ones.

It is acknowledged that alternative methods of adversarial learning exist. Focusing on the modeling of adversarial cognition, we provide only one possible procedure. In particular, optimized algorithms can be adopted for different concrete games [38]. Nevertheless, the process is expected to offer some direction regarding the realization of abstract modeling of games in practical game-playing scenarios.

Several topics remain to be explored in the future. First, to concentrate on the effects of a player’s cognition about their opponents, adversarial cognition is modeled as a one-level reasoning result. Notably, the opponent of a player may also hold an adversarial cognition about this player. Moreover, the player may give further consideration to his opponent’s cognition regarding his cognition, and so on. Therefore, this process represents a kind of high-level cognitive reasoning, which can be explored in future work. Another issue is the dynamic evolution of adversarial cognition. As an illustrating example, the current algorithm for learning the opponent’s cognition is a one-shot process based on the playing history. However, with an increase in observed information about the opponent, more knowledge can be gained, which should lead to a more accurate learning result about the opponent’s cognition. Thus, the online incremental learning of an opponent’s cognition would also be interesting to explore. With a close relation to cognitive theory [39,40], our study also raises concerns about the logical methods of reasoning and verification. This suggests that our framework offers a good platform for theoretical exploration under practical scenarios for a contingent topic on the correlation between logic and game theory.

## Figures and Tables

**Table 1:** Utility for terminal nodes.

Nodes	Overall Utility	Nodes	Overall Utility
$s_{1a}^{2}$	(0.005, 0.995)	$s_{2a}^{2}$	(0.5, 0.5)
$s_{1b}^{2}$	(0.995, 0.005)	$s_{2b}^{2}$	(0.95, 0.05)
$s_{1c}^{2}$	(0.005, 0.995)	$s_{2c}^{2}$	(0.005, 0.995)
$s_{1d}^{2}$	(0.45, 0.55)	$s_{2d}^{2}$	(0.45, 0.55)
$s_{1e}^{2}$	(0.30, 0.70)	$s_{2e}^{2}$	(0.005, 0.995)
$s_{1f}^{2}$	(0.95, 0.05)	$s_{2f}^{2}$	(0.95, 0.05)

## Data Availability

Data are contained within the article.
